# The Shoulder Function Index (SFInX): evaluation of its measurement properties in people recovering from a proximal humeral fracture

**DOI:** 10.1186/s12891-016-1138-0

**Published:** 2016-07-18

**Authors:** Alexander T. M. van de Water, Megan Davidson, Nora Shields, Matthew C. Evans, Nicholas F. Taylor

**Affiliations:** La Trobe Sport Exercise and Rehabilitation (LASER) and Department of Rehabilitation, Nutrition and Sport, School of Allied Health, La Trobe University, Bundoora, Victoria 3086 Australia; Department of Allied Health, Northern Health, Bundoora, Victoria Australia; Melbourne Orthopaedic Group, Windsor, Victoria 3181 Australia; Allied Health Clinical Research Office, Eastern Health, Box Hill, Victoria 3128 Australia

**Keywords:** Shoulder fractures, Rehabilitation, Shoulder function index, Psychometrics, Validity, Reliability, Outcome Assessment (Health Care)

## Abstract

**Background:**

Concerns about test administration, reliability estimations, content and internal structure (dimensionality) of available shoulder measures for people with proximal humeral facture led to the development of a new clinician-observed outcome measure: the Shoulder Function Index (SFInX). The SFInX measures shoulder function by judgement of actual ability to perform daily tasks in which the shoulder is involved. Patients and health professionals had input into the instrument development, and Rasch analysis was used to create a unidimensional, interval-level scale. This study comprehensively evaluated the measurement properties of the SFInX in people recovering from a proximal humeral fracture.

**Methods:**

Data were collected on 92 people [79 women, mean age 63.5 years (SD13.9)] who sustained a proximal humeral fracture within the previous year on three occasions to allow for evaluation of the following measurement properties: construct validity (convergent, discriminant and known-groups validity), longitudinal validity (responsiveness), intra-rater reliability (one week retest interval), and inter-rater reliability (*n* = 20 subgroup; two independent raters). Comparative measures were Constant Score and Disabilities of the Arm Shoulder and Hand (DASH) and discriminative measure was a mental status questionnaire. Minimal clinically important difference, floor and ceiling effects and feasibility of the SFInX were also evaluated. *A priori* hypotheses were formulated where applicable.

**Results:**

Results for construct validity testing supported hypothesised relationships (convergent validity *r* = 0.75–0.89 (Constant Score and DASH); discriminant validity *r* = −0.08 (mental status); known-groups validity *r* = 0.50). For longitudinal validity, lower correlations (*r* = 0.40–0.49) than hypothesised (*r* = 0.50–0.70) were found. The SFInX scores changed more (10.3 points) than other scales, which could indicate that the SFInX is more responsive than the comparative measures. Intra-rater and inter-rater reliability found ICCs of 0.96 (95 % CI 0.94–0.97) and 0.91 (95 % CI 0.63–0.97) respectively, with low measurement error (SEM = 3.9–5.8/100). A change of 11–12 points (out of 100) was indicative of a clinically important difference.

**Conclusions:**

The SFInX is a feasible outcome measure which clinicians can use to reliably measure and detect clinically important changes in the construct of ‘shoulder function’, the ability to perform activities in which the shoulder is involved, in people recovering from a proximal humeral fracture.

## Background

Fractures of the proximal humerus are the third most common limb fracture in older people [1, 2], and the incidence is expected to increase because of the ageing population [3]. A proximal humeral fracture is debilitating for the person directly after the trauma with loss of arm function and severe pain, and often results in ongoing disability with a prolonged period of recovery and rehabilitation [4, 5].

To monitor a patient’s shoulder function during rehabilitation after a proximal humeral fracture, measures with sufficient reliability that can detect change in shoulder function are required. Many different measures related to the shoulder exist [6], varying from single range of motion measurements to multi-item questionnaires focussing on activities, shoulder pain and social participation. However, impairment measures do not necessarily reflect daily functioning of a patient, and subjective measures provide a different insight into the patient’s problem than objective or performance-based measures [7–9]. A systematic review [10] found that psychometric properties of shoulder outcome measures were limited in evaluating people recovering from a proximal humeral fracture. Knowledge is therefore lacking regarding what outcome measures are sufficiently reliable and valid to measure shoulder function, and are able to measure change over time in those patients. A recent study [11] performed a head-to-head psychometric evaluation of five shoulder outcome measures (including Constant Score, Disabilities of the Arm Shoulder and Hand (DASH), Oxford Shoulder Score) and provided some evidence for construct validity and test-retest reliability of these measures, but also highlighted measurement concerns regarding their absolute reliability. For example, the DASH had wide limits of agreement with 15 to 21 out of 100 points difference required to exceed measurement error. The study also highlighted issues with the measures’ content and structure, some of which had also been reported by others [12–14]. For example, multiple measurement dimensions are combined into a single outcome score, subjective and objective measures are combined and lacking of standardisation. Such limitations could influence the quality of measurement, the evaluation of treatment effects and the monitoring of progress of individuals recovering from a proximal humeral fracture.

For these reasons, a new outcome measure for shoulder function was developed: the Shoulder Function Index (SFInX) [15, 16], a clinician-observed outcome measure developed for people with a proximal humeral fracture within the *Activities* domain of the *International Classification of Functioning, Disability and Health* framework [17]. A patient is asked to perform activities, which are judged by a clinician on successful completion. Since it is based on the actual ability to perform tasks in which the shoulder is involved, it is reflective of the daily limitations a person has after a proximal humeral fracture. The SFInX was developed with input from patients in the target population and health professionals. Rasch analysis was used to create an interval-level scale that is unidimensional capturing ‘shoulder function’ as reflected by the tasks [15, 16]. However, further evaluation of measurement properties is required to evaluate the potential for the SFInX to monitor the functional progress of individuals and as an outcome measure in clinical trials of interventions for people with a proximal humeral fracture.

The aim of the current study was to evaluate the measurement properties of the SFInX in people recovering from a proximal humeral fracture. In addition, floor or ceiling effects and clinical feasibility were examined.

## Methods

A prospective longitudinal study to evaluate measurement properties in people with a proximal humeral fracture was conducted. Ethics approval was obtained from two relevant human ethics committees [La Trobe University Human Ethics Committee (FHEC11-113) and Eastern Health Human Ethics Committee (LR86-1011)], and all participants provided written informed consent.

### Participants

Data from participants who took part in the development study of the SFInX [15, 16] were used to evaluate the measurement properties. Clinical testing was performed with a larger set of items (*n* = 19) which were reduced during development to 13 items in the final SFInX [15, 16]. People with a proximal humeral fracture were identified from three metropolitan hospitals (Victoria, Australia). Potentially eligible participants were mailed an invitation to take part in the study, and non-responders were followed up by phone. Interested individuals were screened over the phone against eligibility criteria (Table 1). Eligible individuals were invited to attend one of the recruitment hospitals or, if they preferred, were offered home visits.

**Table 1:** Selection criteria of participants

*Inclusion criteria* - 18 years or older - Isolated proximal humeral fracture, or proximal humeral fracture-dislocation with similar clinical presentation after reduction - Available for recruitment within one year (365 days) post-fracture - Any treatment received for proximal humeral fracture before or during study participation - Ability to complete English-language questionnaires and to follow simple instructions in English - Short Portable Mental Status Questionnaire score 6–10 (indicates intact or mildly impaired cognitive functioning)
*Exclusion criteria* - Other serious medical issues from the trauma (e.g. hip fracture, wrist fracture, nerve lesion, traumatic brain injury, muscle ruptures) - Potentially confounding medical conditions (e.g. hemiplegic arm, previous shoulder surgery, re-fracture, severe rheumatoid arthritis)

Sample sizes were based on requirements for Rasch analysis [18]. As part of the development of the SFInX [15, 16], we aimed to recruit a consecutive sample of n ≥ 75. With a drop out of up to 25 %, a sample size of 56 would still allow for adequate estimation of the intra-rater reliability coefficients with sufficiently precise confidence intervals [19, 20].

A convenience sub-group of participants who attended one of the recruitment sites were also invited to be part of the inter-rater reliability study. Based on calculations from Walter et al. [19], we aimed to recruit a sample of *n* ≥ 19 for estimation of the inter-rater reliability coefficients.

### Measurement protocol and outcome measures

Data were collected on three occasions: an initial assessment within 1 year post-fracture and a follow-up assessment 6 weeks later (Table 2). One week later, a third assessment for intra-rater reliability was performed. This one-week interval was considered sufficient to minimise the clinician’s recall of previous results, and participants’ shoulder function was expected to be stable. Assessment sessions took 30–45 min.

**Table 2:** Overview of measurement protocol and related measurement properties evaluation

	Recruitment	Follow up 1 (6 weeks later)	Follow up 2 (7 weeks later)
General information	Demographic data, QoL		
‘Shoulder function’	DASH, SFInX, Constant Score	DASH, SFInX, Constant Score	DASH, SFInX, Constant Score
Perception of change		‘Overall perception of change’-scale	‘Overall perception of change’-scale
Measurement property	Assessment point		
	Initial	Follow up 1	Follow up 2
Intra-rater reliability SFInX		✓	✓
Inter-rater reliability SFInX^a^	✓	✓	✓
Validity			
Convergent	✓	✓	
Discriminant and Known-groups	✓		
Longitudinal (or responsiveness)	✓	✓	
MCID		✓	

^a^: paired ratings were collected at the same assessment point

With regards to inter-rater reliability study, a second rater conducted a second assessment of the SFInX during one of the visits of a sub-group. The first rater was a physiotherapist with 5 years of clinical experience and was the developer of the SFInX. The second rater was a senior physiotherapist with 20 years of experience in the clinical assessment and treatment of the shoulder. The second rater had no previous experience with the SFInX, was provided with the SFInX manual which included descriptors and scoring instructions, but had no specific training in the SFInX prior to the study. This method was employed since training is usually not given when using an outcome measure described in the literature, and might therefore better reflect the agreement between a novice user of the SFInX (second rater) and a more experienced rater (developer of the SFInX). The assessments by the two raters were conducted in the same clinical environment, a maximum of 15 min apart, and administered in a blinded and independent manner. The two raters administrated the SFInX for this study in random order to control for potential rater effects, such as influence on behaviour or expectations of the participant.

Characteristics of the participants were gathered through interview and completion of short questionnaires regarding the cause of fracture, preferred/dominant side, independent living status [21]. Overall health-related quality of life was measured using the EuroQoL-5D and EuroQoL-VAS [22]. Based on x-rays, fractures were classified by an orthopaedic consultant according to three classifications systems for comprehensive description of fracture types: the Neer [23], AO [24] and Codman-Hertel [25] classifications.

Comparison measures used as part of this measurement properties evaluation were the Disabilities of the Arm, Shoulder and Hand and Constant Score for convergent validity, the Short Portable Mental Status Questionnaire for discriminant validity and a ‘global rating of change’ scale for minimal clinically important difference.

### Shoulder Function IndeX

The SFInX is a 13-item clinician-observed outcome measure that evaluates ‘shoulder function’ [15, 16]. A clinician observes the performance of a patient on each item, and judges, based on category descriptions, whether the tasks were completed successfully. The scoring categories for five items are ‘able’ or ‘unable’, and eight items also have a middle ‘partially able’ category, which is chosen when compensation is used to complete the task. Total raw scores are converted to a 0–100 interval level SFInX score using the conversion table on the assessment form (Appendix). On this converted scale, 0 points means ‘unable to perform any activity successfully to any extent’ and 100 points means ‘able to perform all activities successfully’.

The SFInX was developed for people with a proximal humeral fracture [15, 16]. Patients and clinicians who treat people with a proximal humeral fracture were actively involved in its development including item generation and providing feedback at multiple stages during the development process. This increased the face and content validity of the outcome measure. Rasch analysis was performed on the 13-item SFInX which confirmed a unidimensional structure providing evidence of structural (construct) validity.

### Disabilities of the Arm, Shoulder and Hand (DASH) questionnaire

The DASH [26] is a multidimensional [14] 30-item patient-reported questionnaire evaluating disability of the upper extremity. The Australian version of the DASH was used ([www.dash.iwh.on.ca]). Items relate to daily activities (21 items; ICF *Activities*), symptoms (6 items; ICF *Body functions*) and social/role function (3 items; ICF *Participation* and *Personal Factors*), and participants are asked to reflect on “the past week”. Item scores are on a 5-point ordinal scale. Recommendations of the developers were followed with regards to missing items (maximum of three) [26]. Total scores range from 0 to 100, where 0 indicates “no disability” and 100 “totally disabled”.

Available psychometric information for the DASH in people with a proximal humeral fracture provides some evidence of convergent validity and longitudinal validity (compared with, for example, Oxford Shoulder Score, Constant Score and EuroQol) [11, 21]. Test-retest reliability (ICC_2,1_) was 0.87 (95 % CI 0.53 to 0.97) [11].

### Constant score

The Constant Score [27, 28] is an impairment-focussed shoulder outcome measure that comprises four parts. The components ‘Pain’ (interval level visual analogue scale from 0 to 15 points; ICF *Body Functions*) and ‘Activities of Daily Living function’ (ordinal scales totalling 0 to 20 points; ICF *Body Functions* and *Participation*) are patient-reported evaluating “the last 24 h”, while ‘range of motion’ (ordinal scales totalling 0 to 40 points; ICF *Body Functions*) and ‘strength’ in 90° abduction (interval level scale from 0 to 25 points; ICF *Body Functions*) are clinician-administered. The possible total score range is 0 to 100 points, where 100 indicates ‘normal’ function. The revised protocol including handling of missing data [27] was followed, with the exception that a hand-held dynamometer (Lafayette Manual Muscle Test System) was used for measuring the ‘strength’ component, since it was more feasible and more common in daily practice than an Isobex® machine [27]. Participants were in a sitting position on an armless chair with back support during testing.

Three studies performed psychometric evaluation of the Constant Score in people with a proximal humeral fracture, providing evidence for convergent validity [11, 29, 30], and longitudinal validity [11] (compared with, for example, Oxford Shoulder Score, Neer Score and DASH). Test-retest reliability (ICC_2,1_) was 0.91 (95 % CI 0.53 to 0.97) [11].

### Short portable mental status questionnaire

Discriminant validity testing compared SFInX scores with raw scores of the Short Portable Mental Status Questionnaire [31], which has ten items (each scored as correct or incorrect) that reflect cognitive functioning. Scores range from 0 to 10, with a higher score (more correct answers) indicating better cognitive function. Although none of the patients were excluded based on this criterion, this questionnaire was also used as screening tool for cognitive impairment and therefore participants were required to score 6 or more points (Table 1).

### Global rating of change scale

A 15-point ‘global rating of change’ scale [32] was used as an anchor to determine the minimal clinically important difference (MCID) of the SFInX. The question participants were asked on paper to answer was “Since your last measurement 6 weeks ago, how much change has there been in the function of your fractures shoulder?”. Scoring options ranged from ‘-7 a very great deal worse’ through ‘0 no change’ to ‘+7 a very great deal better’. Interpretation of the scores [33] were: 0 and ±1 were considered ‘no change’, ±2 and ±3 a ‘small change’ and equivalent to the MCID, ±4 and ±5 a ‘moderate change’, and ±6 and ±7 a ‘large change’.

### Data analysis

Statistical Package for Social Sciences version 19 [34] and Microsoft Excel (Microsoft, Redmond, WA, USA) were used for statistical analyses. Data were tested for and fulfilled assumptions for parametric calculations (Shapiro-Wilk test). Descriptive statistics were used to describe sample characteristics. Change scores of the outcome measures between initial assessment and first follow up were calculated and evaluated with paired *t*-tests for significance testing of change and evaluated with the Cohen’s d effect size [35] for head-to-head comparison of change measured by the outcome measures. Psychometric terminology, analyses and reporting of results followed recommendations from recent guidelines and frameworks [36, 37] and the quality assessment checklist from the COSMIN initiative [38, 39]. *A priori* hypotheses were formulated where applicable.

### Reliability and measurement error

#### Intra-rater reliability and inter-rater reliability

Using total SFInX scores, intra-class correlation coefficients (ICC_2,1_) for agreement with 95 % confidence intervals (95 % CI) were calculated as a relative measure of reliability [40–42].

#### Measurement error

As an absolute measure of reliability, several related statistics were used to present estimates of measurement error: Standard Error of Measurement (SEM; SEM = SD_baseline_ * √(1-ICC_2,1_)), Minimal Detectable Change at 95 % confidence (MDC_95;_ MDC_95_ = SEM * √2 * 1.96) and Bland and Altman’s Limits of Agreement (LoA; mean difference ± 1.96*SD_difference_) [43].

#### Inter-rater item-rating agreement

Agreement of item ratings between raters was calculated per item using Cohen’s kappa [44] for dichotomous items, and (quadratic) weighted kappa for polytomous items [45]. In addition, percentage agreement was calculated.

### Construct validity

Construct validity was evaluated as convergent, discriminant and known-groups validity.

*Convergent validity*, or associations between outcome measures aiming to measure the same construct of shoulder function, was evaluated by calculating Pearson’s product–moment correlation coefficient (*r*) between total scores of the SFInX and DASH, and the Constant Score. We hypothesised negative (DASH) and positive (Constant Score) linear correlations of moderate magnitude (*r* = 0.50–0.70), since the comparison measures also include constructs from the ICF *Body Functions* domain. Convergent validity was collected at two time points, at initial assessment and at follow-up assessment 6 weeks later. Evaluation at the second time point was completed because the data were easily collected and to determine if estimates of convergent validity were stable during the clinical course after fracture.

*Discriminant validity*, or the absence of association between outcome measures aiming to measure different constructs, was evaluated by calculating Pearson’s *r* between total scores of the SFInX and Short Portable Mental Status Questionnaire [31]. We hypothesised negligible or weak (*r* = −0.30 to 0.30) correlations.

*Known-groups or extreme groups validity* is a form of validation in which mean scores on an outcome measure are shown to significantly differ between groups that would be expected to differ on the basis of a specific characteristic [36]. We hypothesised significantly lower (independent *t*-test, *p* < 0.05) SFInX scores for people within three months post-fracture than scores for people more than nine months post-fracture. In addition, a moderate positive linear correlation (*r* = 0.40–0.60) was hypothesised between SFInX scores and time post-fracture.

### Longitudinal validity (responsiveness)

To determine the ability to detect change in shoulder function over time, Pearson’s *r* between change scores [38, 39] of the SFInX and DASH, and the Constant Score were calculated. We hypothesised negative (DASH) and positive (Constant Score) linear correlations of moderate magnitude (*r* = 0.50–0.70) between change scores, since the comparison measures also include constructs from the ICF *Body Functions* domain.

### Other characteristics

Two methods were used to determine the minimal clinically important difference (MCID). The anchor-based method used a ‘global rating of change’ scale [32]. Scores of ±2 and ±3 were considered a ‘small change’ and equivalent to the MCID [33]. The distribution-based method followed Norman et al. [46] who proposed half a standard deviation of scores at baseline as a good estimate for the MCID.

Although higher floor or ceiling effect thresholds have been used in studies with people with a proximal humeral fracture [21], we followed McHorney and Tarlov [47] who defined ‘problematic’ as >15 % of the sample receiving the lowest or highest score possible.

Feasibility was evaluated as administration time and equipment required for the SFInX.

## Results

**Fig. 1:**
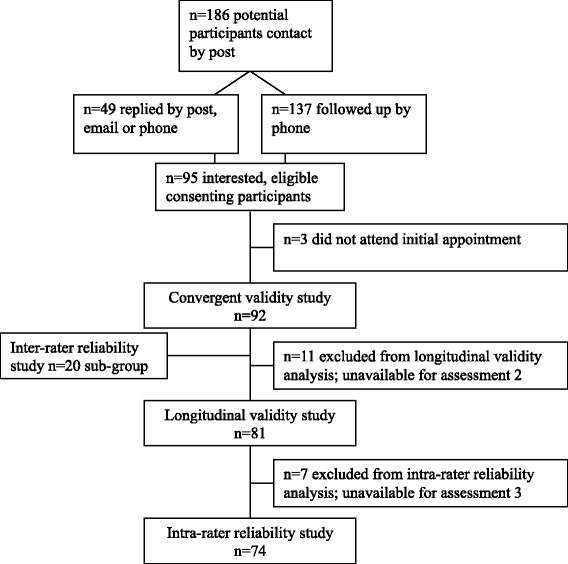
Flowchart of participants in the study

Between February 2012 and January 2013, data were collected on 92 people with a proximal humeral fracture, who were recruited on average 26 weeks (SD15) post-fracture (range 5–52) (Table 3). The cause of fracture was a simple fall in 71 participants (77 %), and a high energy trauma in 21 participants (23 %). Nine people (10 %) had sustained a fracture-dislocation, which after reduction had a similar clinical course to an isolated proximal humeral fracture. Home visits were made for 25 out of the 92 (27 %) initial assessments. Eighty-one participants (88 %) were available for the 6-week follow up measurement (mean 42.4 days, SD 5.9). One week later (mean 6.8 days, SD 1.8) 74 (80 %) participants were re-assessed (Fig. 1). Reasons for loss to follow up were work commitments, holidays or no further interest.

**Table 3:** Baseline characteristics of sample (*n* = 92)

Characteristics		no. (%) or Mean ± SD (range)
Participants		92 (100 %)
men		13 (14 %)
women		79 (86 %)
Age (years)		63.5 ± 13.9 (23–92)
Living situation		
alone		23 (25 %)
with spouse/family		69 (75 %)
EuroQoL		
5D (0–1)		0.68 ± 0.18 (0.15-1.0)
VAS (0–100)		76.5 ± 14.4 (30–100)
Time after fracture (weeks)		26.5 ± 15.1 (5–52)
(¼ yearly distribution)		20, 30, 19, 23
Fracture side		
Right		42 (46 %)
Left		50 (54 %)
Fracture of dominant side		
Yes		44 (48 %)
No		48 (52 %)
Fracture management		
Conservative		74 (80 %)
Surgical	ORIF	16 (17 %)
	Hemi	2 (2 %)
Fracture classifications		no. (%) or fracture type (no.)
AO Classification	A	53 (58 %)
		1.1 (11), 1.2 (4), 1.3 (4)
		2.1(13), 2.2(4), 2.3(9)
		3.1 (2), 3.2 (4), 3.3 (2)
	B	36 (39 %)
		1.1 (23), 1.2 (1)
		2.1 (5), 2.3 (6), 3.2 (1)
	C	3 (3 %)
		1.1 (1), 2.1 (1), 3.2 (1)
Neer Classification	2-part	55 (60 %)
		2FD ant (4) 2GT (15)
		2aSN (22), 2bSN (9), 2cSN (5)
	3-part	35 (38 %)
		3FD ant (2), 3GT (31), 3LT (2)
	4-part	2 (2 %)
		4-part (2)
Hertel Classification		1 (30), 2 (1), 3 (19), 7 (32)
		8 (1), 9 (5), 10 (1), 12 (3)

EuroQoL scores indicated a diminished quality of life at time of recruitment (Table 3). The SFInX, DASH and Constant Score indicated that participants had reduced shoulder function at this time point. Differences in average total scores at the 6-week follow up assessment showed an improvement in shoulder function (Table 4).

**Table 4:** Shoulder function outcome measure scores at recruitment and 6 week follow up (values are mean (SD))

	Recruitment (*n* = 92)	6 week follow up (*n* = 81)	difference in points^a^	Effect size (Cohen’s d)	Paired *t*-test
SFInX v1.0 (0–100)	62.1 (23.4)	71.9 (18.9)	10.3 (14.0)	0.44	*t*(*df*) = 6.62(80), *p* < 0.001
DASH (0–100)^b^	71.6 (21.1)	77.3 (19.1)	6.8 (11.9)	0.31	*t*(*df*) = 5.10(80), *p* < 0.001
Constant (0–100)	52.2 (20.2)	60.4 (18.3)	9.0 (10.4)	0.44	*t*(*df*) = 7.81(80), *p* < 0.001

^a^Data from *n*=81 of whom data from two assessments were available, was used to calculate the difference in points and effect sizes, and used to perform the paired *t*-test

*SFInX v1.0*, Shoulder Function IndeX version 1.0

*DASH*, Disabilities of Arm, Shoulder and Hand questionnaire (^b^scores have been reversed to facilitate comparison of total scores; 100 points indicates “no disability”)

*Constant*, Constant Score

## Measurement properties of the SFInX

### Reliability and measurement error

**Fig. 2:**
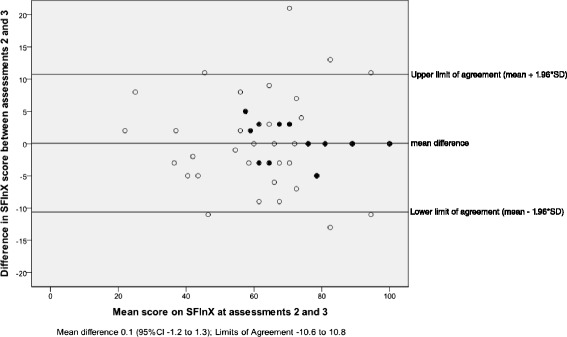
Bland and Altman-plot with 95 % Limits of Agreement for SFInX total score absolute agreement between retest sessions (Assessment 2 and 3; full black dots represent change scores of two or more participants)

Data for intra-rater reliability were available from 74 participants who completed both 6 and 7 week follow up assessments. The ICC_2,1_ for agreement was 0.96 (95 % CI 0.94 to 0.97). The SEM was 3.9 points (out of 100) and MDC_95_ was 10.8 points. Figure 2 shows the Bland and Altman-plot providing the mean difference (0.1 points, SD5.5) between assessments and the Limits of Agreement (−10.6 to 10.8). As can be seen in Fig. 2, these data included one outlier who increased their score by 21 points when retested one week later.

**Fig. 3:**
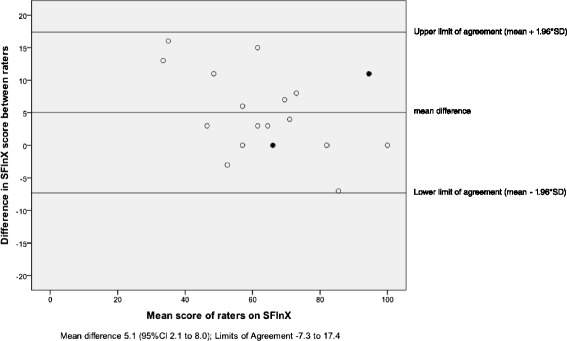
Bland and Altman-plot with 95 % Limits of Agreement for SFInX total score absolute agreement between raters (full black dots represent change scores of two participants)

Data for inter-rater reliability from a sub-group of 20 participants yielded an ICC_2,1_ for agreement of 0.91 (95 % CI 0.63 to 0.97). Total scores between raters were compared with Bland and Altman-analysis (mean difference 5.1 (SD6.3), Limits of Agreement −7.3 to 17.4; random error estimation 12.3). Figure 3 shows the significant systematic difference of 5.1 points (95 % CI 2.1 to 8.0; *t*(df) = 3.59(19), *p* < 0.01).

Agreement of category ratings between raters ranged from k = 0.42 to 1.00 and percentage agreement of 40–100 % (Table 5). Item 3 ‘washing back of the opposite shoulder’ was the item with the most different ratings. Rater 2 rated 12 out of 20 participants higher than Rater 1 on item 3, and agreed on the remaining eight. The difference in scoring on this item was responsible for the low kappa and systematic difference found with the Bland and Altman-analyses. Wording of category descriptions was subsequently altered in the final 13-item version of the SFInX to aim for higher inter-rater reliability and inter-rater item-rating agreement.

**Table 5:** Intra-rater and inter-rater reliability and measurement error estimates of the SFInX

				Bland-Altman analysis	
Reliability	ICC_2,1_ (95 % CI)	SEM	MDC_95_	Mean difference (95 % CI)	Limits of Agreement
Intra-rater	0.96 (0.94 to 0.97)	3.9	10.8	0.1 (−1.2 to 1.3)	−10.6 to 10.8
Inter-rater	0.91 (0.63 to 0.97)	5.8	16.1	5.1 (2.1 to 8.0)	−7.3 to 17.4
	Kappa	%			
Inter-rater item agreement	0.42 to 1.00	40-100 %			
Dichotomous items (*n* = 5)	0.62 to 1.00	90-100 %			
Polytomous items (*n* = 8)	0.42 to 0.92	40-95 %			

*ICC* Intra-class correlation coefficient, *CI* Confidence Interval, *MDC* Minimal Detectable Change, *SEM* Standard Error of Measurement

### Construct validity

Providing evidence of convergent validity, correlations of the SFInX scores with the DASH and Constant Score at baseline (*n* = 92) were −0.78 and 0.89 (*p* < 0.01) respectively, which were somewhat higher than hypothesised. At the 6-week follow up assessment (*n* = 81) the correlations were −0.75 and 0.87 (*p* < 0.01), respectively.

Discriminant validity testing confirmed the hypothesis of no correlation (*r* = −0.08, *p* = 0.44) between the SFInX and Short Portable Mental Status Questionnaire.

Known-groups validity was evaluated by comparing initial SFInX scores between people ≤3 months post-fracture (*n* = 21; mean SFInX score 41.4, SD21.1) and those ≥9 months post-fracture (*n* = 23; mean SFInX score 75.1, SD20.5). This difference of 33.7 points was significant (95 % CI 21.0 to 46.3; *t* = −5.37, *p* < 0.01). Also, a significant moderate correlation (*r* = 0.50, *p* < 0.01) between time post-fracture and total SFInX score was found.

### Longitudinal validity (responsiveness)

Analyses of longitudinal validity found weaker correlations between change scores (difference between initial and 6 week follow-up) than hypothesised. SFInX change scores correlated with changes in the DASH (*r* = −0.40, *p* < 0.01) and the Constant Score (*r* = 0.49, *p* < 0.01). Table 4 shows the SFInX scores changed more (10.3 points), relative to the scale width of 100, than the DASH (6.8/100 points) or Constant Score (9.0/100 points), which could mean that the SFInX is more responsive to change in ‘shoulder function’ than the other measures. A subgroup of patients (*n* = 20) that was recruited ≤3 months post-fracture and reassessed 6 weeks later, was analysed and found similar correlations between change scores (*r* = 0.43–0.46) to those reported in the full sample. Also in this subgroup the SFInX had the largest mean change scores.

### Other characteristics

The anchor-based and distribution-based methods used to estimate the MCID for improvement provided similar values. Using the average SFInX score difference of people reporting a ‘small change’ (*n* = 21), the anchor-based MCID was 10.3 points (out of 100). The distribution-based MCID was 11.7 points (half the SD_baseline_ of 23.2). The MCID for deterioration was not calculated since only 2 of the 81 participants (2.5 %) reported more limitations from the shoulder at the 6-weeks follow up assessment.

Using the cut-off percentage of >15 %, the SFInX did not show a problematic floor or ceiling effect. At recruitment 2 of 92 participants (2 %) received the lowest and 10 participants (11 %) the highest SFInX score possible. Six weeks later no participant had the lowest score possible (0 %), and 12 out of 81 (14.8 %) participants had the highest possible score at the second assessment. Seven of the 12 had the highest score at both assessments.

Time to complete the 13-item SFInX is estimated at 5 to 7 min once the tester is familiar with the test administration. Training to use the SFInX for health professionals who manage patients with a proximal fracture of the humerus is not required. However, familiarisation should include reading the SFInX manual for item descriptions and decision rules. During clinical testing (247 assessments) we used a 19-item development version of the SFInX (mean 8 min 7 s [(SD1min 51 s; range 2 min to 14 min 13 s]), which still included removed items that took longer to complete, such as walking and turning/rolling in bed [15, 16]. A SFInX assessment will therefore be shorter on average, but also depend on the ability and mobility of the individual patient.

Several objects were required to make testing functional and simulate daily activities: a cup, objects of 1.5, 3 and 6 kg, a (soccer) ball and a (shopping) bag. Suggestions for objects have been made in the SFInX manual [15], (http://sfinx.blogs.latrobe.edu.au/)].

## Discussion

This study provided evidence of measurement properties for the SFInX as a feasible and reliable outcome measure of ‘shoulder function’, which is capable of detecting clinically important changes in ‘shoulder function’ of people recovering from a proximal humeral fracture. This study provides evidence for the construct validity of the SFInX in the form of convergent, discriminant and known-groups validity testing.

The SFInX was developed as a clinician-observed outcome measure (COOM). This type of administration is new in shoulder function measurement and has benefits over other types of outcome administration. For example, available performance-based shoulder measures such as the FIT-HaNSA and simple shoulder endurance test [48, 49] focus mainly on endurance (timing and specific weights) and are limited clinically and functionally by not covering a range of tasks. Also, clinician-administered measures such as the Constant Score [27, 28] and American Shoulder and Elbow Surgeons’ Examination Scale [50] have limitations as outcome measure. They combine patient-reported and clinician-administered components, incorporate multiple domains of functioning into a single score [11], and have arbitrary scorings to their components [12]. Such content and structural issues may raise concerns about accurate reflection of shoulder function and may not yield a score that is easily interpretable.

Patient-reported outcome measures (PROMs) are widely used and greatly valued. However, PROMs are influenced by several factors. For example, outcomes of PROMs focussing on ‘function’ have been strongly associated with perceived levels of pain compared with actual function [9, 51], and provide information on patient perception rather than actual ability or physical performance [7, 8, 52]. Also, factors such as anxiety and fear-avoidance might influence self-reported physical function [53]. PROMs may provide different information to a person’s performance ability. It is therefore advised to use the SFInX as a COOM of actual ability and well developed PROMs together for comprehensive measurement of shoulder function.

Reliability analysis showed that for both the intra-rater and inter-rater reliability ICCs were over 0.90, which is considered good for use in groups, for example at an organisational level or in research projects, and in individual patients [36, 54]. This was confirmed by a low SEM of 3.9 out of 100 points. The MDC_95_ informs that we can be 95 % sure that a change of 10.8 SFInX points or more exceeds measurement error. These values are similar when compared to another unidimensional clinician-observed outcome measure: the de Morton Mobility Index [55]. For other shoulder measures such as the DASH and Constant Score SEMs of 6.5 and 4.5 out of 100 points have previously been found in people with a proximal humeral fracture [11] which indicate MDC_95_ values of 18 and 12.5 points respectively. From the inter-rater reliability study a MDC_95_ of 16 points was found, which suggests that when a second rater would evaluate a patient’s shoulder function with the SFInX, a difference of 16 points would exceed measurement error. However, this number included the systematic error between raters of 5 points due to item 3. Therefore, the error estimate of 12.3 points might be a more accurate indicator for measurement error between raters when item 3 would be more consistently scored or when systematic error is taken into account. Comparing the MCID of 10.3 (anchor-based method) or 11.7 (distribution-based method) points with the MDC_95_ (10.8 points), it can be suggested that a difference in score of 11–12 points can be considered a clinically important change that exceeds measurement error. Although some argue that the distribution-based method of MCID is more related to minimal detectable change than MCID [56], it is an accepted method for MCID estimation [36]. In addition, the MCID is a variable concept depending on baseline scores, directions of change and methods used. Therefore, different methods were used to estimate the MCID and MCID values were interpreted as an estimated range of scores required to be considered clinically important.

The design and measurement properties of the SFInX indicate that it can be used to monitor a person’s ‘shoulder function’ from as early as first use of the arm until independent performance of daily tasks for self-care and around the house. Validated in a sample of people from five weeks up to one year post-fracture and with a large range of abilities, the SFInX may be valuable for early measurement during rehabilitation, monitoring progress in patients and as a potential indicator for discharge from health care services such as physical therapy.

Similarly, the SFInX can be used as an evaluative instrument in clinical research investigating the clinical management in people with a proximal humeral fracture. High-quality evidence and treatment guidelines are currently lacking [57–59], indicating that randomised controlled trials evaluating management strategies in this population are required. Functional outcomes measured by well-developed measures such as the clinician-observed SFInX, should be used.

### Limitations

Some limitations in the study require consideration. The low agreement between raters on item 3 ‘washing the back of the opposite shoulder’ (k = 0.42) may have negatively influenced the reliability estimations of the SFInX. The item’s category descriptions may have contributed to the different ratings and were altered after analysis. The final 13-item SFInX (Appendix) [15], (http://sfinx.blogs.latrobe.edu.au/), contains the re-worded category descriptions for item 3. Although inter-rater agreement and reliability showed improvement when re-analysed with data from item 3 assumed equal, future reliability testing with the revised category descriptions is required.

The small sample size of the inter-rater reliability study can be seen in light of preliminary estimations. It is recommended to have sample sizes of approximately 50 patients for estimations with smaller confidence intervals [39]. Future studies with multiple raters in clinical settings or video recordings are needed to confirm the estimations from this smaller inter-rater reliability study.

The study sample varied with regards to time after fracture at study inclusion. Although this allowed for variety in shoulder function for the development of the SFInX, future prospective studies could concentrate on following up patients from admission to discharge while also recording more details on treatment than recorded in the current study. This could benefit further analyses on longitudinal validity (responsiveness), MCID and discharge predictions of the SFInX in a homogenous sample in relation to the clinical course. Additionally, further Rasch analyses could be carried out with stratification of patients at different stages of healing after fracture to confirm the SFInX as a unidimensional and invariant scale for this population.

## Conclusion

The new Shoulder Function Index is sufficiently reliable and is valid for clinicians to monitor shoulder function of individuals and groups of people with a proximal humeral fracture. With its construct of measuring shoulder function as the ability to perform activities in which the shoulder is involved, it can now be used as an evaluative outcome measure in clinical and research settings.

## Abbreviations

SFInX, Shoulder Function Index; DASH, Disabilities of the Arm, Shoulder and Hand; MCID, Minimal Clinically Important Difference; ICC, Intra-class Correlation Coefficient; SEM, Standard Error of Measurement; MDC_95,_ Minimal Detectable Change at 95 % confidence; LoA, Limits of Agreement; COOM, Clinician-observed outcome measure; PROMs, Patient-reported outcome measures
